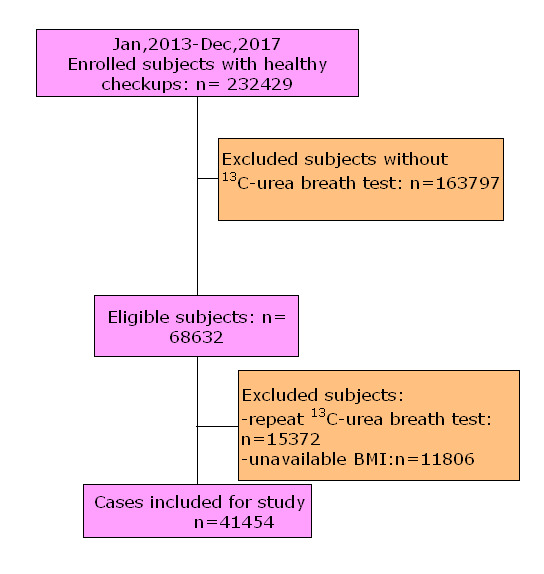# Association between body mass index and Helicobacter pylori infection in asymptomatic individuals: A cross-sectional study

**DOI:** 10.1097/MD.0000000000049133

**Published:** 2026-06-19

**Authors:** Xiaohong Zhu, Qianchun Wang, Xueqin Bao, Simon Stock, Xiuli Dong, Maddalena Zippi, Zarrin Basharat, Wandong Hong

**Affiliations:** a Department of Anesthesia, The First Affiliated Hospital of Wenzhou Medical University, Wenzhou, Zhejiang, People’s Republic of China; b Department of Gastroenterology and Hepatology, The First Affiliated Hospital of Wenzhou Medical University, Wenzhou, Zhejiang, People’s Republic of China; c Department of Gastroenterology and Hepatology, The First Affiliated Hospital of Wenzhou Medical University, Wenzhou, Zhejiang, People’s Republic of China; d Department of Surgery, World Mate Emergency Hospital, Battambang, Cambodia; e Department of Gastroenterology and Hepatology, The First Affiliated Hospital of Wenzhou Medical University, Wenzhou, Zhejiang, People’s Republic of China; f Unit of Gastroenterology and Digestive Endoscopy, Sandro Pertini Hospital, Rome, Italy; g Jamil-ur-Rahman Center for Genome Research, Dr. Panjwani Center for Molecular Medicine and Drug Research, International Center for Chemical and Biological Sciences, University of Karachi, Karachi, Pakistan; h Department of Gastroenterology and Hepatology, The First Affiliated Hospital of Wenzhou Medical University, Wenzhou, Zhejiang, People’s Republic of China.

**Keywords:** age, body mass index, epidemiology, Helicobacter pylori, obesity

## Abstract

We delineated the relationship between body mass index (BMI) and incidences of Helicobacter pylori infection in overweight (obese) and normal weight asymptomatic individuals. This cross-sectional study involved participants who had undergone health checkups for H. pylori infections between January 2013 and December 2017. The association between gender, age, BMI and H. pylori infection prevalence was investigated. In total, 41,454 subjects were enrolled in this study. The overall H. pylori infection prevalence was 42.5%, 48.0%, 50.7% and 54.9% in under weight, normal weight, pre-obese, and obese individuals, respectively. Pre-obesity/obesity (OR = 1.15; 95%CI 1.10 to 1.20; *P* < .001) was correlated with increased H. pylori infection prevalence compared to subjects with normal weight. Increased BMI (OR = 1.04; 95%CI 1.03 to 1.06; *P* < .001) was also correlated with increased H. pylori infection prevalence compared to individuals with normal weight. However, Subgroup analysis indicated that there are no relationship between BMI and H. pylori infection prevalence in individuals with age < 30 among all subjects (OR = 1.01; 95%CI 0.99 to 1.03; *P* = .282). Increased BMI is correlated with increased H. pylori infection prevalence among individuals with age of 30 or more years.

## 1. Introduction

H. pylori is a pathogen that is transmitted from human to human.^[1]^ Although the majority of infected people remain asymptomatic, it causes chronic active gastritis in all the subjects that it colonizes. Therefore, H. pylori presence is considered an infection irrespective of an individual’s symptoms and stage of disease.^[2]^ In many developing countries, its prevalence is high, which is attributed to socioeconomic status and hygiene levels.^[3]^

Obesity has become a health problem of global concern and its relationship with H. pylori has been investigated. However, data reporting on the correlation between obesity and H. pylori are inconclusive. Xu et al indicated that body mass index (BMI) was markedly and positively correlated with H. pylori infection, and a high BMI correlated with increased infection risk. Suki et al documented that among symptomatic patients, H. pylori presence was positively correlated with overweight and obesity. Chen et al found that H. pylori infected individuals aged <50 years have an increased risk of being obese, relative to those without this infection.^[4]^ On the other hand, Lender et al suggested that there is an inverse association between obesity rate and H. pylori prevalence in developed countries.^[5]^ The association between H. pylori infection and BMI in asymptomatic subjects with normal weight has not been reported previously.

Thus, we compared the prevalence of H. pylori infection between pre-obese/obesity and subjects with normal weight. Moreover, we assessed the association between prevalence of H. pylori and BMI in asymptomatic subjects with normal weight.

## 2. Materials and methods

### 2.1. Study design and subject selection

All asymptomatic subjects who had undergone annual routine health checkups between January 2013 and December 2017 were included in this cross-sectional study. Assessment of H. pylori infection was based on the ^13^C-urea breath test after a minimum 6-hour fast.^[6,7]^ The urea breath test can noninvasively and accurately detect H. pylori infection.^[8]^ It has a sensitivity of 95.9%, specificity of 95.7% for diagnosis of H. pylori infection.^[9]^ Exclusion criteria were repeat tests for the same individual between January 2013 and December 2017 (only the first test was included in this study), and inaccessibility of findings for breath tests, weight and height. Age, gender, and findings from breath tests were documented. BMI was determined by weight (kg) divided by height (m) squared. The overall H. pylori infection prevalence was determined as: (all positive H. pylori testcases)/ (all cases who had been subjected to the H. pylori test).

### 2.2. Statistical analysis

Categorical variables were expressed by counts and proportions and compared by the χ2 test. Assessment of continuous data for normal distribution was performed using the Shapiro–Wilk test. Based on findings from the Shapiro–Wilk test, continuous variables were presented by mean ± standard deviation or median and interquartile range (IQR). The independent-samples t-test or the Kruskal-Wallis nonparametric test were used to compare the variables.^[10]^

Linear trends of continuous and categorical variables were respectively assessed using a nonparametric Wilcoxon rank sum test and a Royston extension of the Cochran-Armitage test.^[11,12]^ When performing linear trend analysis, BMI was grouped into 4 categories: <18.5 kg/m^2^ (under weight), 18.5 to 24.9 kg/m^2^(normal weight), 25 to 29.9 kg/m^2^(overweight or pre-obese), and equal to or more than 30 kg/m^2^ (obese).

Logistic regression analysis was performed to assess the association between BMI and H. pylori infection. odds ratios were determined at 95% confidence interval (CI) according to logistic regression analysis. Subgroup analysis of H. pylori prevalence by age groups and sex was performed. Age was allocated into 5 subgroups: ≤30 years, 31 to 40 years, 41 to 50 years, 51 to 60 years, ≥61 years. Two-sided *P*-values below .05 denoted significance. Participants with missing data on breath test results, weight, or height were excluded from the analysis, as stated in the exclusion criteria; therefore, no additional imputation methods were required. Multicollinearity among independent variables (age, sex, and BMI) was assessed by examining the variance inflation factor variance inflation factor (VIF), and all variance inflation factor values were below 2, indicating no significant multicollinearity. STATA version 12.0 was used for analyses.

## 3. Results

### 3.1. Baseline characteristics of participants

**Figure 1.**
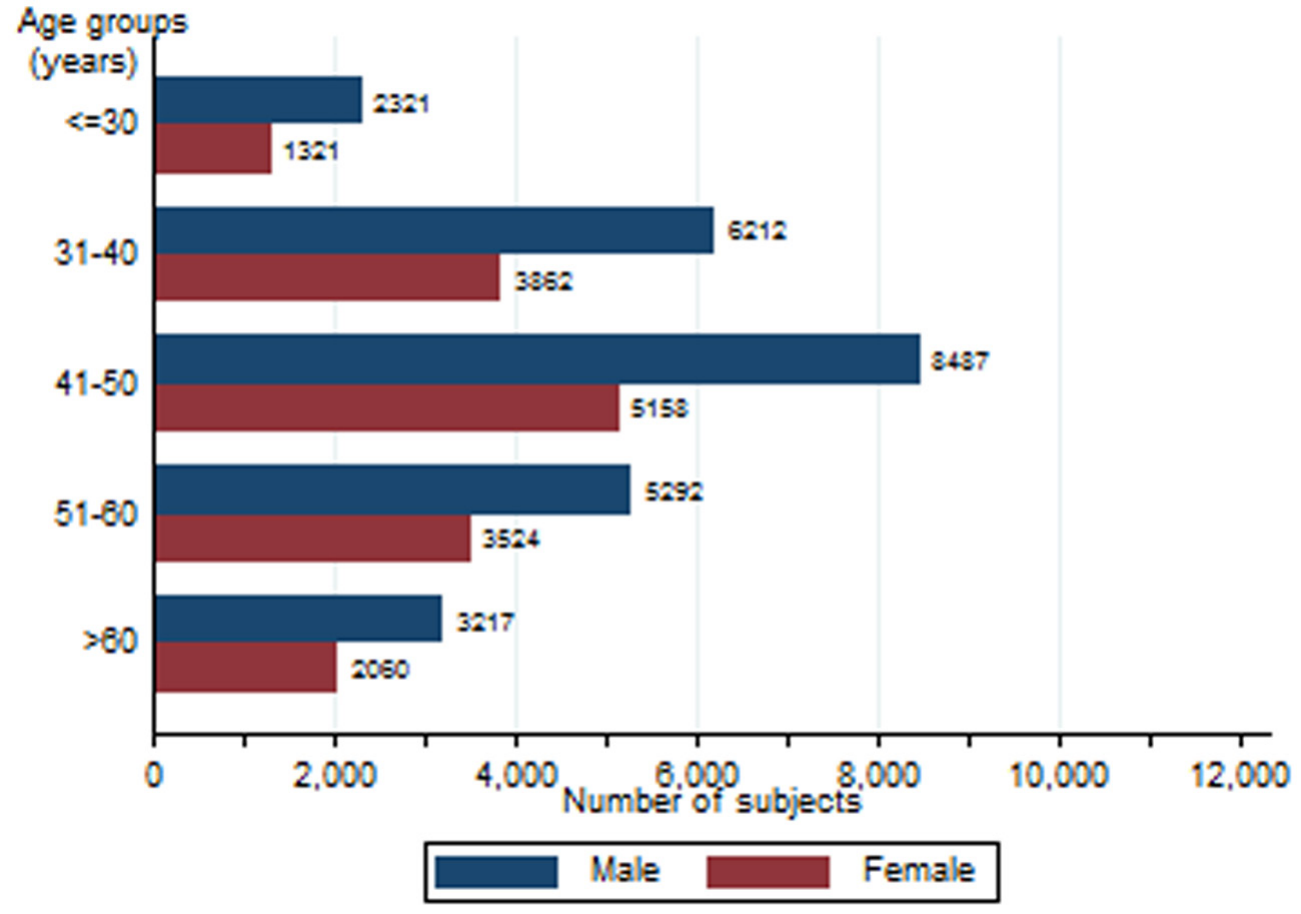
Distribution of sex and age groups among 41,454 subjects irrespective of survey year.

The baseline characteristics of the study participants are summarized in Table 1. Of the 41,454 participants, 25,529 (61.6%) were male and 15,925 (38.4%) were female, averaging 46.3 ± 11.8 years (Supplementary Fig. 1). H. pylori infection prevalence was 48.4%. Compared to those without, individuals with H. pylori were likely to be older (mean age: 46.6 ± 11.5 vs 46.0 ± 12.1, *P* <.001), with women forming a higher percentage (39.0% vs 37.8%, *P* = .01). Figure 1 shows the distribution of age groups and sex among 41,454 subjects. There were 1,496 (3.61%) underweight subjects, 26,107 (62.98%) had a normal weight, 12,502 (30.16%) were pre-obese, while13,49 (3.25%) were obese.

**Table 1:** Baseline characteristics of study participants.

Characteristics	Total (N = 41,454)	H. pylori positive (N = 20072)	H. pylori negative (N = 21382)	*P*-value
Age years (mean ± SD)	46.3 ± 11.8	46.6 ± 11.5	46.0 ± 12.1	<.001
Sex n (%)				
Male	25,529 (61.6%)	12,291 (61.2%)	13,238 (61.9%)	.01
Female	15,925 (38.4%)	7781 (38.8%)	8144 (38.1%)	
Age group n (%)				
≤30 yr	4986 (12.0%)	2145 (43.0%)	2841 (57.0%)	<.001
31–40 yr	9132 (22.0%)	4338 (47.5%)	4794 (52.5%)	
41–50 yr	12,436 (30.0%)	6093 (49.0%)	6343 (51.0%)	
51–60 yr	9960 (24.0%)	4960 (49.8%)	5000 (50.2%)	
≥61 yr	4940 (12.0%)	2536 (51.3%)	2404 (48.7%)	
BMI category n (%)				
Underweight (< 18.5 kg/m^2^)	1496 (3.61%)	636 (42.5%)	860 (57.5%)	<.001†
Normal weight (18.5–24.9 kg/m^2^)	26,107 (62.98%)	12,531 (48.0%)	13,576 (52.0%)	
Pre-obese (25.0–29.9 kg/m^2^)	12,502 (30.16%)	6338 (50.7%)	6164 (49.3%)	
Obese (≥ 30 kg/m^2^)	1349 (3.25%)	741 (54.9%)	608 (45.1%)	
Overall *H. pylori* prevalence	48.4%	—	—	–

—, Data not available in the original manuscript; to be supplemented by the authors.

BMI = body mass index, H. pylori = Helicobacter pylori, SD = standard deviation.

† Percentages represent proportion within H. pylori positive or negative group.

‡ Percentages represent H. pylori infection prevalence within each BMI category.

* Test for trend.

### 3.2. Pre-obesity, obesity, and H. pylori prevalence

Of the 41,454 participants, the overall H. pylori infection prevalence was 42.5%, 48.0%, 50.7%, and 54.9% in underweight, normal weight, pre-obese, and obese individuals, respectively. This implied a linear relationship between H. pylori infection prevalence and BMI (Test for trend: *P* < .001).

When performing logistic regression analysis, BMI was used as a categorical variable and was divided into 3 classifications: underweight, normal weight, and pre-obesity or obesity. Our data indicated that pre-obesity or obesity (OR = 1.15; 95%CI 1.10 to 1.20; *P* < .001) was correlated with increased H. pylori infection prevalence compared to subjects having normal weight (after adjustment for sex and age). In other words, in pre-obese/obese individuals, their odds of getting H. pylori infection are 1.15 times greater than individuals with normal weight (holding sex and age constant). Underweight individuals (OR = 0.81; 95%CI 0.73 to 0.90; *P* <.001) had a lower risk of developing H. pylori infection compared to subjects with normal weight after adjustment with respect to sex and age.

**Figure 2.**
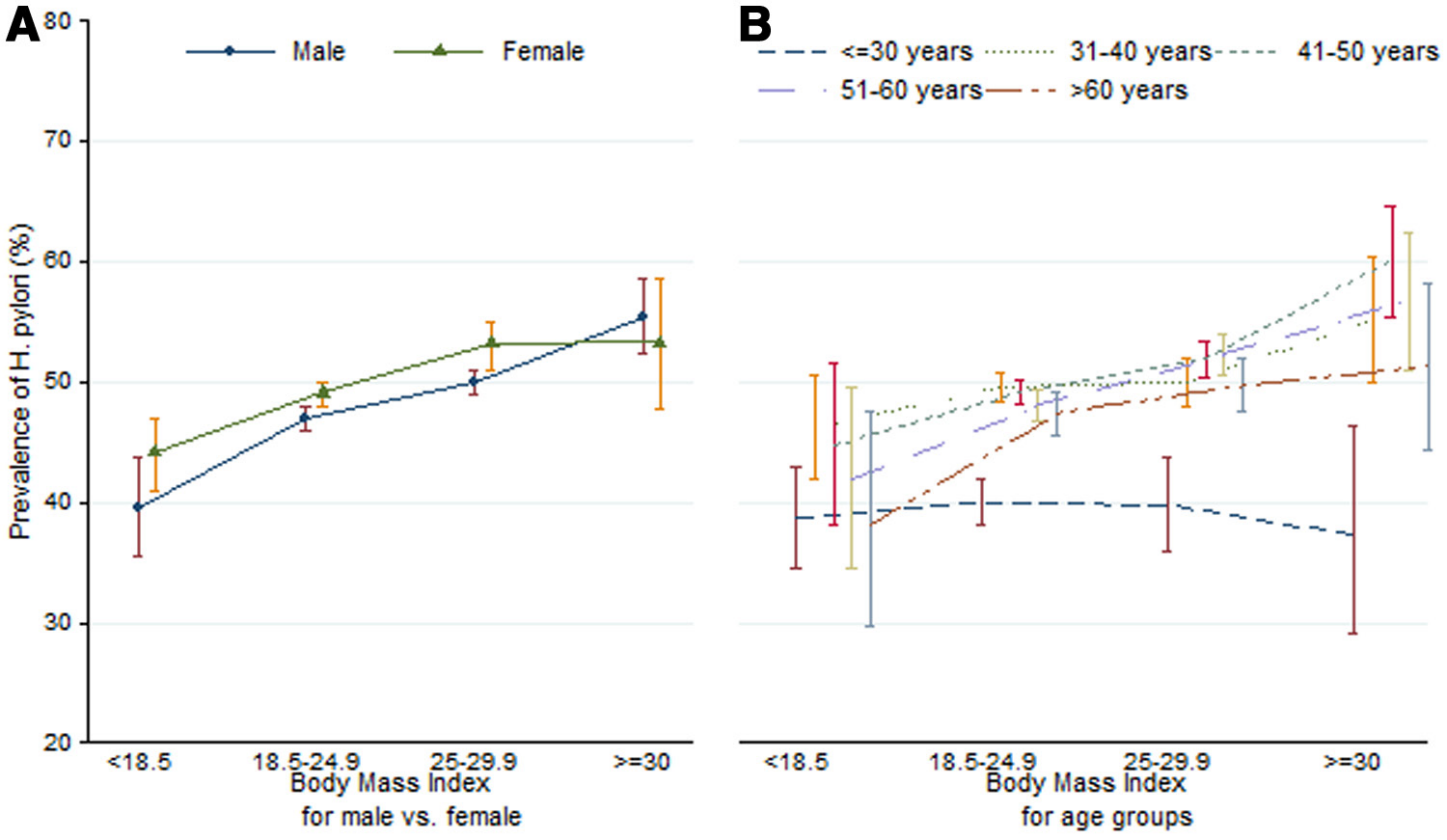
Prevalence of Helicobacter pylori infection in different BMIs, stratified by gender (A) and age groups (B). Data are expressed as prevalence and its 95% CI. Data were analyzed for all 41,454 individuals. CI = confidence interval.

**Figure 3.**
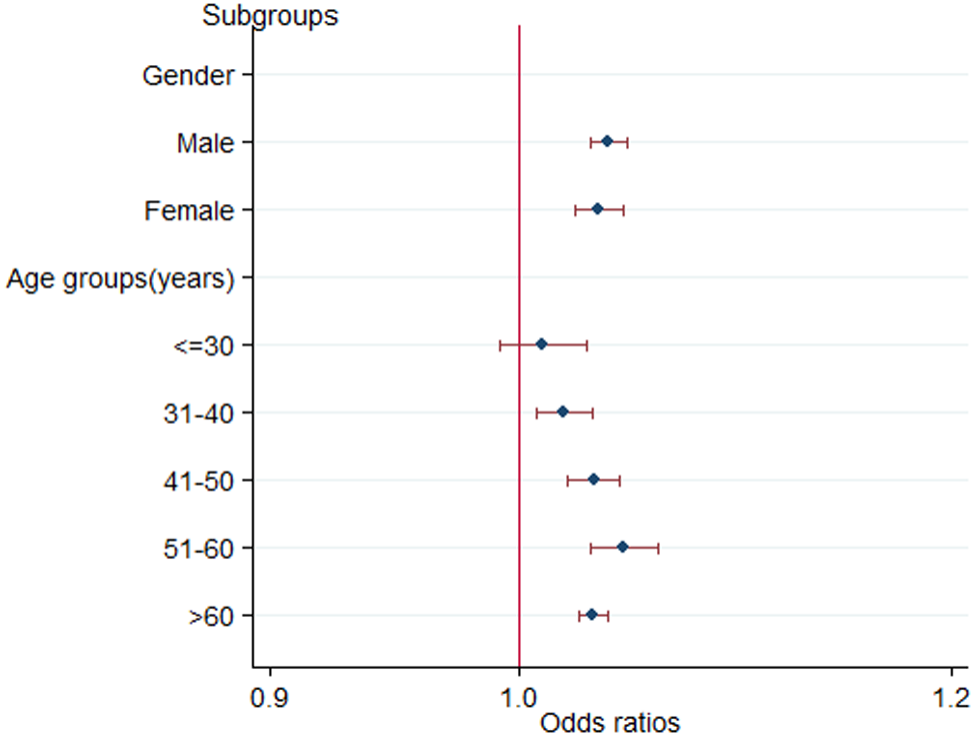
Logistic regression analysis of the relationship between all subjects and H. pylori infection in different gender and age groups. The forest plot represents odds ratios and 95% CIs. The red vertical line is the reference line of odds ratio, i.e., having a value of 1. A 95% CI spanning across 1 is not statistically significant. The overlap of the red vertical line and dash (95% confidence interval of odds ratio) means that the relationship between BMI and prevalence in a subgroup did not reach statistical significance. An odds ratio >1 in a subgroup indicates an increased occurrence of H. pylori infection with increased BMI (it describes a positive relationship). BMI = body mass index, CI = confidence interval.

Subgroup analysis indicated that individuals in the age group >30 as well as pre-obese or obese individuals had a high H. pylori infection prevalence, relative to subjects with normal weight, irrespective of gender (Fig. 2). No relationship was inferred between BMI and H. pylori infection prevalence in individuals in the age group < 30 (OR = 1.01; 95%CI 0.99 to 1.03; *P* = .282) (Fig. 3).

### 3.3. Normal weight and H. pylori

There were 26,107 individuals with normal weight. When performing logistic regression analysis, BMI was used as a continuous variable. Our data suggested that increased BMI (OR = 1.04; 95%CI 1.03 to 1.06; *P* <.001) in subjects with normal weight was still correlated with increased H. pylori infection prevalence after adjusting for sex and age.

**Figure 4.**
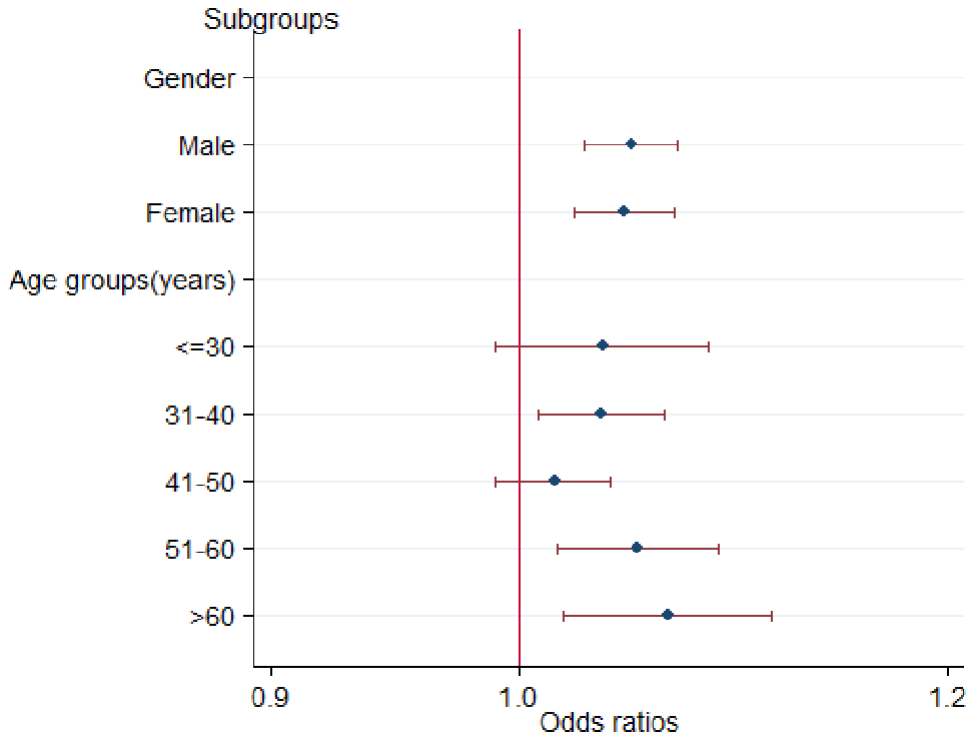
Logistic regression analysis of H. pylori infection in different gender and age groups for subjects having normal weight (26,107 individuals). The forest plot denotes odds ratios and 95% CIs. The red vertical line, with a value of 1, is the reference line of odds ratio. A 95% CI spanning across 1 is not statistically significant. Overlap among the red vertical line and horizontal dash means the relationship between BMI and prevalence in a subgroup did not reach statistical significance, while an odds ratio >1 for a subgroup indicates an increased occurrence of H. pylori infection with an increase in BMI (depicting a positive relationship). BMI = body mass index, CI = confidence interval.

Subgroup analysis showed BMI association with the prevalence of H. pylori infection with differences in gender and age grouping (31–40, 51–60 and >60 years) (Fig. 4). However, there was no relationship between BMI and H. pylori infection prevalence in individuals that were part of age group <30 (OR = 1.04; 95%CI 0.99 to 1.08; *P* = .125 or that of age group 41–50 years (OR = 1.02; 95%CI 0.99 to 1.04; *P* = .239) (Fig. 4).

## 4. Discussion

H. pylori infection prevalence varies among races and countries. With improving socio-economic states and hygiene, H. pylori infection rates exhibit decreasing trends in many regions.^[13]^ In this study, the H. pylori infection prevalence was 48.4%, higher than the study by Xu et al (43.8%) on Chinese individuals. It was significantly lower than that of the Israeli people, as reported by Suki et al (61.3%). These variations in H. pylori prevalence are likely due to the levels of industrialization of society, urbanization, hygiene status, sanitation, accessibility to clean water, ethnicity, racial differences, as well as varied socioeconomic status.

The relationship between BMI and H. pylori has not been conclusively determined. Xu et al suggested that H. pylori infection is not correlated with pre-obesity/obesity in a retrospective study on Chinese people.^[14]^ Salles et al^[15]^ documented that H. pylori might be the reason behind undernutrition and weight loss in some patients older than 75 years. Wu et al^[16]^ conducted a case-control study and showed an inverse association between H. pylori seropositivity and morbid obesity. Vo et al^[17]^ suggested that in symptomatic children, H. pylori infection decreases the prevalence of obesity. However, Lee et al reported that individuals with a high BMI (≥25.0) exhibited a high H. pylori seropositivity relative to those with a normal BMI (18.5–23.0) (OR 1.17, 95% CI: 1.00–1.37).^[18]^ Xu et al^[19]^ reported that overweight/obese participants had a high H. pylori infection prevalence relative to that of lean participants, and BMI exhibited a positive linear association with H. pylori infection prevalence. Suki et al^[20]^ also documented a positive relationship between H. pylori infection and obesity in symptomatic patients. Our study suggests a linear association between H. pylori infection prevalence and BMI (Test for trend: P < .001). Logistic regression analysis showed that pre-obesity/obesity (OR = 1.15; 95%CI 1.10 to 1.20; P < .001) was correlated with increased H. pylori infection prevalence when compared to subjects with normal weight after adjustment for sex and age. This difference in results might be due to different study designs and sample sizes, variations in the recruitment of participants, and different detection methods utilized for H. pylori infection. Serology, which can give false positive results due to past infections, may also result in an overestimation of the H. pylori infection rate. In addition, it is assumed that symptomatic patients may have a poorer appetite than asymptomatic individuals, which may affect the incidence of obesity reported in different studies. Our results also indicate that in asymptomatic subjects with normal weight, increased BMI (OR = 1.04; 95%CI 1.03 to 1.06; *P* <.001) is correlated with increased H. pylori infection prevalence. Suki et al^[20]^ reported that within the normal weight range, the symptomatic patients with a high-normal BMI had a high H. pylori positivity relative to those with low-normal BMIs.

The mechanisms by which pre-obesity/obesity have a higher H. pylori infection prevalence relative to subjects with normal weight are unclear. One reason might be an imbalance in hormones, such as leptin and ghrelin. Ghrelin, an orexigenic hormone, is primarily secreted from the stomach,^[21]^ with various physiological functions, such as the stimulation of appetite, growth hormone release, and fat accumulation.^[22]^ It also has cardioprotective, antiapoptotic, vasodilatory, immune modulatory, and anti-oxidative functions. Its roles are facilitated via its receptor (growth hormone secretagogue receptor), with both the hormone and receptor having vital roles in age-related obesity, type 2 diabetes (T2D), and insulin resistance.^[23]^ It was reported that fasting ghrelin levels are low in obese individuals and are markedly associated with BMI and insulin resistance.^[24]^ The plasma ghrelin levels for H. pylori-positive individuals are markedly lower than those of H. pylori-negative controls.^[25]^ Ghrelin has anti-inflammatory effects and is a promising agent for the treatment of injury and inflammatory diseases.^[26]^ Dysregulated synthesis of ghrelin in the H. pylori-colonized stomach has been proposed as a new clue for the pathogenesis of H. pylori-associated gastric inflammation.^[27]^

Our subgroup analysis results suggested the absence of any relationship between BMI and H. pylori infection prevalence in individuals in the age group <30, either among all 41,454 individuals (Figs. 2 and 3) or among 26,107 individuals with normal weights (Fig. 4), which suggests that mechanisms of pre- obesity/obesity between adolescents and adults may differ. There is a strong persistence of severe obesity from adolescence to young adulthood.^[28]^ Weight gain from young to middle adulthood may be correlated with high risks of several types and lead to mortality.^[29]^ Leptin is an adipocyte-secreted hormone.^[30]^ Stylianou et al reported that BMI and body fat are positively correlated with leptin and negatively correlated with ghrelin levels in adolescents.^[31]^ Leptin could treat obesity caused by congenital leptin deficiency through leptin replacement. This could be applied in the treatment of asymptomatic obese individuals having H. pylori infection, and we have a future vision of applying this treatment to our patients.

The strong points of our study include a large sample size that gives the study sufficient statistical power. All subjects had routine health checkups, and all H. pylori infections were diagnosed by urea breath test, which makes our study homogeneous. In addition, this is the first study to evaluate the association between the prevalence of H. pylori infection and BMI in asymptomatic healthy individuals with normal weight. Our study does have some limitations. First, we used BMI ≥ 30 kg/m^2^ as a surrogate for obesity because percentage body fat was not available. Despite the good association between percentage body fat and BMI, the accuracy of using BMI for obesity diagnosis is limited, especially for persons in intermediate BMI ranges. A ≥ 30 kg/m^2^ cutoff for BMI has good specificity but misses over half of individuals with excess fat.^[32]^ Body fat measurement is a better approach for assessing obesity in people whose BMI is below 30 kg/m2.^[33]^ Thus, our evaluation of obesity in this study may not have been optimal. Second, the association between BMI and percentage body fat is linked with ethnicity. Asians differ from Caucasians and from each other in terms of their BMI/percentage body fat relationship.^[34]^ Therefore, it may not be possible to generalize our results to other populations around the globe. Third, similar to several previous epidemiological analyses of H. pylori,^[35–37]^ our study only enrolled a few variables (absence or presence of H. pylori, BMI, age, and gender); we did not record additional information such as medical history, dietary habits, or any other parameters through questionnaires, gastric disease, and a history of eradication. Fourth, as this is a cross-sectional study, causality cannot be inferred from our findings; the observed associations should be interpreted as correlations rather than causal relationships. Therefore, one must interpret our results with caution, and it will be vital to assess these factors in the future.

## 5. Conclusion

Pre-obesity/obesity was correlated with increased H. pylori infection compared to subjects with normal weight. In asymptomatic subjects with normal weight, increased BMI was still associated with increased H. pylori infection prevalence. However, there was no association between BMI and H. pylori infection in asymptomatic young individuals aged < 30 years. Future longitudinal studies are warranted to explore the causal pathways underlying this association, and mechanistic studies investigating the roles of metabolic hormones such as ghrelin and leptin may provide further insights.

## Author contributions

**Data curation:** Xueqin Bao, Zarrin Basharat.

**Formal analysis:** Xueqin Bao, Simon Stock, Xiuli Dong.

**Methodology:** Xiuli Dong, Maddalena Zippi.

**Software:** Qianchun Wang.

**Supervision:** Qianchun Wang.

**Validation:** Wandong Hong.

**Writing – original draft:** Xiaohong Zhu.

**Writing – review & editing:** Wandong Hong.

**Figure:**